# EHMTI-0281. Frequency of headache in stroke

**DOI:** 10.1186/1129-2377-15-S1-C66

**Published:** 2014-09-18

**Authors:** N Zaric, N Milovanovic-Kovacevic, M Savic

**Affiliations:** 1 Neurosonology, Hospital for Cerebrovascular Diseases Sveti Sava, Belgrade, Serbia

## Background

Headache is a relatively common symptom associated with various types of stroke. It belongs to symptomatic headaches and is classifed in the 6th group IHS classification.

The aim of this study was to compare the frequency of the headache in stroke patients depending on sex, age, type of stroke, duration of headache, location and size of the cerebral lesion.

## Methods

From January 1 to December 31,2013. 5,476 stroke patients were admitted in St. Sava hospital. Out of them, 843 patients were treated, female 402, male 441, mean age 67,9. The patients were studied using the standard protocol including CT and MR. The presence of headache was established by taking history from the patients or relatives.

## Results

Among these patients, 48.9% had experienced headache, with higher frequency in female (64.3%), less than 70 years old. Frequency of headache in patients with ischemia was 41.6%, and with hemorrhage 50.3%. Headache was most common in patients with cortical lesions (56.7%) and with lesions located in the basilar artery (41.3%). Large lesions (>2 cm) were more frequently followed with headache (62.3% vs. 28%). The average duration of headache in ischaemic stroke was 25 hours compared to 65 hours in haemorrhagic stroke.

## Conclusions

Headache was more frequent in females (P < 0,01), in patients with hemorrhage (P < 0,05), with large ischemic lesions (P < 0,01), located in the basilar distribution (P < 0,05) and in the cortical area (P < 0,05).

No conflict of interest.

